# Tratamiento con toxina botulínica en un entorno de recursos limitados: experiencias de una institución pública

**DOI:** 10.7705/biomedica.7383

**Published:** 2025-08-11

**Authors:** Gloria Andrea Panesso, Juan Diego Martínez, Annelise Velasco, Stefanía Forero, Daniel Perdomo, Ángela Villamil, Edna Rocío González, Claudio Alejandro Jiménez

**Affiliations:** 1 Departamento de Farmacología, Unidad de Servicios de Salud Simón Bolívar, Subred Integrada de Servicios de Salud Norte E. S. E., Bogotá, D. C., Colombia Unidad de Servicios de Salud Simón Bolívar Unidad de Servicios de Salud Simón Bolívar Subred Integrada de Servicios de Salud Norte E. S. E. Bogotá, D. C. Colombia; 2 Departamento de Farmacología Clínica, Fundación Universitaria de Ciencias de la Salud, Bogotá, D. C., Colombia Fundación Universitaria de Ciencias de la Salud Departamento de Farmacología Clínica Fundación Universitaria de Ciencias de la Salud Bogotá, D. C. Colombia; 3 Departamento de Neurología, Unidad de Servicios de Salud Simón Bolívar, Subred Integrada de Servicios de Salud Norte E. S. E., Bogotá, D. C., Colombia Unidad de Servicios de Salud Simón Bolívar Unidad de Servicios de Salud Simón Bolívar Subred Integrada de Servicios de Salud Norte E. S. E. Bogotá, D. C. Colombia

**Keywords:** toxinas botulínicas, usos terapéuticos, interacciones farmacológicas, países en desarrollo, accesibilidad a los servicios de salud., Botulinum toxins, therapeutic uses, drug interactions, developing countries, health services accessibility.

## Abstract

**Introducción.:**

La toxina botulínica se destaca por sus múltiples aplicaciones terapéuticas; tiene más de 30 indicaciones reconocidas en distintas especialidades. Sin embargo, a pesar de los estudios realizados en Latinoamérica, no hay una caracterización integral sobre su amplio espectro de uso clínico.

**Objetivo.:**

Clasificar el uso farmacoterapéutico de la toxina botulínica en un centro de salud público de Bogotá, para identificar los riesgos de interacciones farmacológicas con otros compuestos y las posibles barreras para su uso. Se pretende fomentar una mejor comprensión de las indicaciones, prácticas y regulaciones de la toxina botulínica en el contexto colombiano.

**Materiales y métodos.:**

Se realizó un estudio transversal en el que se analizó la administración de la toxina botulínica mediante la metodología de prescripción-indicación. Se recopiló información de las historias clínicas y del sistema de prescripción electrónica “Mi prescripción”. Para el análisis de los datos, se utilizó el *software* estadístico JAMOVI, versión 2.2.5.

**Resultados.:**

De 197 pacientes tratados con la toxina botulínica, la mayoría de las prescripciones se registraron bajo la categoría de trastornos neurológicos (70,6 %), aunque la indicación más frecuente correspondió a alteraciones de la articulación temporomandibular (18,8 %). En niños, la indicación predominante fue la parálisis cerebral espástica (90 %). El 92,4 % de las aplicaciones clínicas coincidieron con las aprobadas por el Instituto Nacional de Vigilancia de Medicamentos y Alimentos de Colombia. Se identificaron riesgos de interacciones farmacológicas en el 30,9 % de los casos, asociadas significativamente con una carga anticolinérgica (p < 0,001).

**Conclusión.:**

La toxina botulínica es ampliamente utilizada en Colombia debido a sus diversas indicaciones clínicas. En general, se encontró una buena correspondencia entre las prescripciones y las recomendaciones de las guías reguladoras. Es crucial evaluar los antecedentes farmacológicos de los pacientes para minimizar el riesgo de interacciones farmacológicas. Se subraya la necesidad de revisar y ajustar la normativa sobre el uso de la toxina botulínica en el país.

## Abstract (en)

**Introduction.:**

Botulinum toxins have at least 30 therapeutic indications across different medical specialties. Some Latin American studies have described the experience of applying botulinum toxin in isolated clinical contexts. However, researchers have yet to provide comprehensive analysis about its use across diverse clinical indications.

**Objective.:**

To classify the therapeutic use of botulinum toxin in a public health center in Bogotá, identifying the risks of pharmacological interactions and possible barriers related to its use. This knowledge will promote a better understanding of the indications, practices, and regulations regarding the use of the botulinum toxin in the Colombian context.

**Materials and methods.:**

A cross-sectional study was conducted to analyze the use of botulinum toxin in a public institution in Bogotá, Colombia, applying the prescription- indication methodology. Sociodemographic, clinical, and pharmacoepidemiologic data were extracted from medical records and the electronic prescription database *Mi prescripción*. A bivariate statistical analysis was performed using JAMOVI software, version 2.2.5.

**Results.:**

A total of 197 patients received a prescription for botulinum toxin. Although the main indication was temporomandibular joint disorder, 70.6% of the prescriptions were classified under neurological conditions. In pediatric patients, the main indication was spastic cerebral palsy (90%). Most described clinical uses (92.4%) were aligned with the indications approved by the *Instituto Nacional de Vigilancia de Medicamentos y Alimentos de Colombia*. Risks of drug interactions were identified in 30.9% of the cases and were significantly associated with an anticholinergic load (p < 0.001).

**Conclusions.:**

Botulinum toxin is used for multiple indications in multiple medical specialties. There is consistency between prescription and indication of the medication recommended by regulatory entities. Reviewing patient’s pharmacological history is essential before prescribing botulinum toxin to reduce the risk of drug interactions.

La toxina botulínica es una proteína producida por bacterias de la familia *Clostridium* spp. Su administración intramuscular genera una parálisis química reversible de las fibras al bloquear la liberación de acetilcolina en la unión neuromuscular ^1^. En 1989, la *Food and Drug Administration* (FDA) aprobó su uso para el tratamiento del estrabismo. Desde entonces, se han creado once formulaciones de toxina botulínica, seis de ellas aprobadas por la FDA ^1^^,^^2^ y, al menos, 30 indicaciones terapéuticas en diferentes especialidades médicas ^2^^,^^3^.

Si bien la toxina botulínica es utilizada ampliamente en la medicina estética para reducir las líneas de expresión causadas por la hiperactividad muscular y el envejecimiento, también tiene otras indicaciones en diferentes especialidades médicas. En neurología, se utiliza para el tratamiento de distonías focales, espasmos hemifaciales y espasticidad; en oftalmología, para estrabismo y nistagmos; y en urología, para la hiperactividad del músculo detrusor y la incontinencia urinaria. Además, se ha demostrado su eficacia en el manejo de la migraña crónica, el dolor neuropático y la sialorrea, lo que refleja su amplio potencial terapéutico (Du X, Patel D. Use of botulinum toxin in the treatment of neuropathic pain: A case series (P10-13.009). Neurology. 2022;98(Suppl. 18). Abstracts: AAN 74^th^ Annual Meeting, Seattle, May 03, 2022. https://doi.org/10.1212/WNL.98.18_supplement.864 ) ^2^^-^^5^.

En cuanto a las indicaciones terapéuticas no estéticas, existen cuatro formulaciones diferentes de la toxina botulínica aprobadas por la FDA para diversas enfermedades: Botox^®^ para el tratamiento de blefaroespasmo, espasmo hemifacial, estrabismo, distonía cervical, migraña, espasticidad de miembros superiores e inferiores (estos últimos en adultos), hiperactividad neurogénica del músculo detrusor, vejiga hiperactiva y arrugas de la frente; Xeomin^®^ como opción terapéutica para blefaroespasmo, distonía cervical, líneas de expresión, espasticidad de miembros superiores y sialorrea en adultos; Dysport^®^ para la distonía cervical, líneas de expresión y arrugas, espasticidad de miembros superiores en adultos y espasticidad de miembros inferiores en general; y Myobloc/Neurobloc^®^ para tratar la distonía cervical ^1^.

En Colombia, el Instituto Nacional de Vigilancia de Medicamentos y Alimentos (Invima) es la institución gubernamental responsable de emitir las aprobaciones de los medicamentos y los registros sanitarios en el país ^6^^,^^7^. Las preparaciones activas de la toxina botulínica aprobadas por el Invima en Colombia para indicaciones no estéticas, incluyen: Magnion®, Botox^®^, Dysport^®^, Neuronox^®^, Siax^®^ y Xeomin^® (^^8^. Sus registros Invima se muestran en el cuadro 1.

**Cuadro 1:** Indicaciones aprobadas de la toxina botulínica de tipo Acón registro INVIMA vigente o en trámite de renovación en Colombia (junio 2021)

Marca registrada	Indicaciones aprobadas	Registro INVIMA
BOTOX® B	Tratamiento de la hiperactividad muscular, por su acción como agente inhibidor de la liberación de acetilcolina INVIMA presináptica, en las siguientes enfermedades: 2016M-0011586-R1 Oftalmológicas: blefaroespasmo esencial benigno o asociado con distonía, estrabismo y distonía focal Neurológicas: parálisis cerebral, tremor, espasticidad, distonías, mioclonías, espasmo hemifacial, cefalea tensional, tortícolis espasmódica Urológicas: hiperactividad del músculo detrusor de la vejiga Otorrinolaringológicas: temblor palatal esencial, disfonía espasmódica Dermatológicas: hiperhidrosis focal axilar y palmar; tratamiento de líneas faciales hiperfuncionales Traumatológicas-ortopédicas: padecimientos espásticos, dolor en espalda, cuello y espina dorsal asociados con contracturas patológicas. Alternativo en la profilaxis del dolor de cabeza en migraña crónica	INVIMA 2016M-0011586-R1
BOTOX®	Tratamiento de la hiperactividad muscular en las siguientes enfermedades: Oftalmológicas: blefaroespasmo esencial benigno o asociado a distonía, estrabismo y distonía focal Neurológicas: coadyuvante o alternativo en parálisis cerebral, tremor esencial que no ha respondido a otros tratamientos orales, espasticidad, distonías, mioclonías que cursen con fenómenos distónicos, espasmo hemifacial, cefalea tensional, tortícolis espasmódica Urológicas: hiperactividad del músculo detrusor de la vejiga Otorrinolaringológicas: temblor palatal esencial, disfonía espasmódica Dermatológicas: hiperhidrosis refractaria a tratamientos convencionales Traumatológicas-ortopédicas: coadyuvante en padecimientos espásticos, dolor de cuello y espina dorsal asociado con contracturas patológicas resistentes a otras medidas terapéuticas, y bruxismo temporomaxilar Proctológicas: fisura anal Gastroenterológicas: acalasia en casos en los que no puede hacerse dilatación neumática o cirugía, tratamiento de líneas faciales hiperfuncionales por médicos entrenados en el uso correcto del producto Alternativo en la profilaxis del dolor de cabeza en migraña crónica	INVIMA 2014M-014172-R2
BOTOX® 50 UI	Tratamiento de la hiperactividad muscular, por su acción como agente inhibidor de liberación de acetilcolina presináptica, en las siguientes enfermedades: Oftalmológicas: blefaroespasmo esencial benigno o asociado a distonía, estrabismo y distonía focal Neurológicas: parálisis cerebral, tremor espasticidad, distonías, mioclonías, espasmo hemifacial, cefalea tensional y tortícolis espasmódica Urológicas: hiperactividad del músculo detrusor de la vejiga Otorrinolaringológicas: tremor palatal esencial, disfonía espasmódica Dermatológicas: hiperhidrosis resistente a tratamientos convencionales, tratamiento de líneas faciales hiperfuncionales Traumatológicas-ortopédicas: padecimientos espásticos, dolor en espalda, cuello y espina dorsal asociados con contracturas patológicas Alternativo en la profilaxis del dolor de cabeza en migraña crónica	INVIMA 2009M-0009951
DYSPORT® 500 UI	Tratamiento sintomático de la espasticidad focal de: INVIMA Extremidades superiores e inferiores en adultos, estas últimas afectadas en la articulación del tobillo como 2020M-0000761-R2 secuela de un accidente cerebrovascular o una lesión cerebral traumática Deformidad dinámica del pie equino o extremidades superiores afectadas en pacientes pediátricos ambulatorios, con parálisis cerebral, mayores de dos años. Tratamiento sintomático de distonía cervical, blefaroespasmo, espasmo hemifacial, hiperhidrosis primaria grave de la axila que no mejora con el tratamiento tópico con antitranspirantes o antihidróticos, hiperhidrosis palmar Mejoría temporal en la apariencia de líneas glabelares (líneas verticales entre las cejas visibles al fruncir el ceño) y líneas cantales laterales (“patas de gallo”, visibles con la máxima sonrisa), moderadas a graves, en adultos menores de 65 años, cuando la gravedad de estas líneas tiene un impacto psicológico significativo en el paciente. El uso de esta intervención no está relacionado con la recuperación o mantenimiento de la capacidad funcional o vital de las personas.	INVIMA 2020M-0000761-R2
DYSPORT® 300 UI	Nuevas indicaciones: tratamiento sintomático de la espasticidad focal de extremidades superiores en adultos o deformidad dinámica del pie equino en pacientes pediátricos ambulatorios, con parálisis cerebral, mayores de dos años Indicado en adultos para el tratamiento sintomático de tortícolis espasmódica o blefaroespasmo, espasmo hemifacial, hiperhidrosis primaria grave de la axila que no mejora con el tratamiento tópico con antitranspirantes o antihidróticos e hiperhidrosis palmar Mejoría temporal en la apariencia de líneas glabelares (líneas verticales entre las cejas visibles al fruncir el ceño) y líneas cantales laterales (“patas de gallo” visibles con la máxima sonrisa), moderadas a graves, en adultos menores de 65 años, cuando la gravedad de estas líneas tiene un impacto psicológico significativo en el paciente.	INVIMA 2019M-0013175-R1
XEOMIN® LD50 (50 UI)	Tratamiento de la hiperactividad muscular, por su acción como agente inhibidor de la liberación de acetilcolina presináptica, en las siguientes enfermedades: Oftalmológicas: blefaroespasmo esencial benigno o asociado a distonía, estrabismo y distonía focal Neurológicas: parálisis cerebral, tremor, espasticidad, distonías, mioclonías, espasmo hemifacial, cefalea tensional y tortícolis espasmódica Urológicas: hiperactividad del músculo detrusor de la vejiga Otorrinolaringológicas: temblor palatal esencial y disfonía espasmódica Dermatológicas: hiperhidrosis focal axilar y palmar, y tratamiento de líneas faciales hiperfuncionales Traumatológicas-ortopédicas: padecimientos espásticos, dolor en espalda, cuello y espina dorsal asociados con contracturas. Secuelas de ataque de espasticidad de las extremidades superiores en adultos	INVIMA 2015M-0016055
XEOMIN® LD 50 (100 UI)	Tratamiento de la hiperactividad muscular, por su acción como agente inhibidor de la liberación de acetilcolina presináptica, en la siguientes enfermedades: Oftalmológicas: blefaroespasmo esencial benigno o asociado a distonía, estrabismo y distonía focal Neurológicas: parálisis cerebral, tremor, espasticidad, distonías, mioclonías, espasmo hemifacial, cefalea tensional y tortícolis espasmódica Urológicas: hiperactividad del músculo detrusor de la vejiga Otorrinolaringológicas: temblor palatal esencial y disfonía espasmódica Dermatológicas: hiperhidrosis focal axilar y palmar, y tratamiento de líneas faciales hiperfuncionales Traumatológicas-ortopédicas: padecimientos espásticos, dolor en espalda, cuello y espina dorsal asociados con contracturas. Secuelas de ataque de espasticidad de las extremidades superiores en adultos.	INVIMA 2015M-0016054
NEURONOX® 200 UI	Tratamiento de la hiperactividad muscular, por su acción como agente inhibidor de la liberación de acetilcolina presináptica, en las siguientes enfermedades: Oftalmológicas: blefaroespasmo esencial benigno o asociado a distonía, estrabismo y distonía focal Neurológicas: parálisis cerebral, tremor, espasticidad, distonías, mioclonías, espasmo hemifacial, cefalea tensional, tortícolis espasmódica Urológicas: hiperactividad del músculo detrusor de la vejiga Otorrinolaringológicas: temblor palatal esencial y disfonía espasmódica Dermatológicas: hiperhidrosis focal axilar y palmar, tratamiento de líneas faciales hiperfuncionales (no relacionado con la recuperación o mantenimiento de la capacidad funcional o vital de las personas) Traumatológicas-ortopédicas: padecimientos espásticos, dolor en espalda, cuello y espina dorsal asociados con contracturas patológicas Alternativo en la profilaxis del dolor de cabeza en migraña crónica	INVIMA 2019M-0019123
NEURONOX® 100 UI	Tratamiento de la hiperactividad muscular en las siguientes enfermedades: Oftalmológicas: blefaroespasmo esencial benigno o asociado con distonía, estrabismo y distonía focal Neurológicas: coadyuvante o alternativo en parálisis cerebral, tremor esencial que no ha respondido a otros tratamientos orales, espasticidad, distonías, mioclonías que cursen con fenómenos distónicos, espasmo hemifacial, cefalea tensional y tortícolis espasmódica Urológicas: hiperactividad del músculo detrusor de la vejiga Otorrinolaringológicas: temblor palatal esencial y disfonía espasmódica Dermatológicas: hiperhidrosis refractaria a tratamientos convencionales y tratamiento de líneas faciales hiperfuncionales Traumatológicas-ortopédicas: coadyuvante en padecimientos espásticos, dolor de cuello y espina dorsal asociado con contracturas patológicas que no han respondido a ninguna otra medida terapéutica, y bruxismo temporomaxilar Proctológicas: fisura anal Gastroenterológicas: acalasia en casos en los que no puede hacerse dilatación neumática o cirugía Alternativo en la profilaxis del dolor de cabeza en migraña crónica	
NEURONOX® 50 UI	Tratamiento de blefaroespasmo esencial benigno en pacientes mayores de 18 años Indicado para el tratamiento de la deformidad del pie equino debido a la espasticidad en pacientes pediátricos con parálisis cerebral mayores de dos años Mejora temporal de las arrugas glabelares, moderadas a graves, asociadas con la actividad del músculo corrugador o prócer en adultos entre 18 y 65 años	INVIMA 2014M-0015013
SIAX® 100 U	Tratamiento de distonías, blefaroespasmo, espasmo hemifacial, estrabismo hiperhidrosis y tratamiento de líneas hiperfuncionales de la región facial Indicado para el tratamiento de las siguientes enfermedades: Oftalmológicas: blefaroespasmo esencial benigno o asociado con distonía, estrabismo y distonía focal Neurológicas: coadyuvante o alternativo en parálisis cerebral, tremor esencial que no ha mejorado con otros tratamientos orales, espasticidad, distonías, mioclonías que cursen con fenómenos distónicos, espasmo hemifacial, cefalea tensional y tortícolis espasmódica Urológicas: hiperactividad del músculo detrusor de la vejiga Otorrinolaringológicas: temblor palatal esencial y disfonía espasmódica Dermatológicas: hiperhidrosis; coadyuvante en padecimientos espásticos, dolor de cuello y espina dorsal asociado con contracturas patológicas resistentes a otras medidas terapéuticas, y bruxismo temporomaxilar Proctológicas: fisura anal Gastroenterológicas: acalasia en casos en los que no puede hacerse dilatación neumática o cirugía	INVIMA 2009M-0009223
SIAX® 50 U	Tratamiento de blefaroespasmo esencial benigno en pacientes mayores de 18 años Tratamiento de la deformidad de pie equino por espasticidad en pacientes pediátricos, con parálisis cerebral, mayores de dos años Mejoramiento temporal de la apariencia de líneas glabelares, moderadas a graves, asociadas con las actividades del músculo corrugador o prócer en adultos entre 18 y 65 años	INVIMA 2015M-0016537
MAGNION 50 UI	Tratamiento de la hiperactividad muscular, por su acción como agente inhibidor de la liberación de acetilcolina presináptica, en las siguientes enfermedades: Oftalmológicas: blefaroespasmo esencial benigno o asociado con distonía, estrabismo y distonía focal Neurológicas: coadyuvante o alternativo en parálisis cerebral, tremor esencial que no ha mejorado con otros tratamientos orales, espasticidad, distonías, mioclonías que cursen con fenómenos distónicos, espasmo hemifacial, cefalea tensional y tortícolis espasmódica Urológicas: hiperactividad del músculo detrusor de la vejiga Otorrinolaringológicas: temblor palatal esencial y disfonía espasmódica Dermatológicas: hiperhidrosis focal axilar y palmar, y tratamiento de líneas faciales hiperfuncionales Traumatológicas-ortopédicas: coadyuvante en padecimientos espásticos, dolor de cuello y espina dorsal asociado con contracturas patológicas resistente a otras medidas terapéuticas, y bruxismo temporomaxilar Proctológicas: fisura anal Gastroenterológicas: acalasia en casos en los que no puede hacerse dilatación neumática o cirugía	NVIMA 2014M-0015326

UI: unidades internacionales

Por otra parte, existen múltiples reportes que respaldan el uso de la toxina botulínica para indicaciones no aprobadas (*off-label*) ^9^^,^^10^. Entre ellas, se encuentran: el dolor posterior a accidentes cerebrovasculares, la neuralgia posherpética, el bruxismo, la hipertrofia maseterina, y el dolor neuropático, miofascial o vesical, entre otros. La carencia de aprobación por parte de instituciones reguladoras de medicamentos, parcialmente debida a la discordancia entre los diagnósticos registrados ante el Invima y aquellos utilizados en la práctica clínica (Clasificación Internacional de Enfermedades, 10^a^ edición, CIE-10), hace que el uso de la toxina botulínica en estos casos sea controvertido.

Aunque hay estudios en Latinoamérica que documentan la experiencia de la toxina botulínica en contextos clínicos específicos e indicaciones puntuales ^11^^-^^13^, hasta la fecha no existe una caracterización integral de su uso en diferentes indicaciones, especialidades médicas e instituciones con recursos limitados, donde su disponibilidad es escasa.

Algunos factores que limitan la disponibilidad de la toxina botulínica en las instituciones prestadoras de servicios de salud podrían estar relacionados con una subestimación del cálculo de la unidad de pago por capitación destinada a esta intervención. Esta subestimación puede deberse a insuficiencias en el control y la vigilancia del uso de la toxina y, por lo tanto, su demanda real en este contexto. Lo anterior resulta en una asignación ineficiente de los recursos financieros destinados al acceso y la distribución de la toxina botulínica, y en un incremento de los gastos de los colombianos que buscan alternativas fuera del sistema de salud ^14^.

Además, en Colombia, el proceso para la prescripción de medicamentos suele estar sujeto a las indicaciones aprobadas por el Invima, y, por lo general, las que están fuera de la ficha técnica (según los diagnósticos disponibles en el CIE-10), tienen un gran riesgo de no ser cubiertas por las aseguradoras, lo que limita aún más el acceso a la toxina botulínica en el sistema de salud público del país.

Por lo tanto, el objetivo de este estudio fue, en primer lugar, describir el perfil de los usos terapéuticos de la toxina botulínica en una institución de salud de la Red Pública Distrital de Bogotá, en la cual existen algunas limitaciones para acceder a estos compuestos; y, en segundo lugar, explorar los posibles riesgos de interacciones farmacológicas y la carga anticolinérgica para proporcionar una visión completa del uso de la toxina botulínica en Colombia. Esto incluye enfatizar en la importancia de modificar las directrices que regulan su uso.

## Materiales y métodos

Este estudio corresponde a un análisis farmacoepidemiológico que utilizó un enfoque de prescripción-indicación dentro de un diseño observacional transversal. Se recopiló información retrospectiva sobre la prescripción de la toxina botulínica a partir de la base de datos “Mi prescripción” (MIPRES), correspondiente a pacientes atendidos en las instituciones públicas de salud de la Subred Integrada de Servicios de Salud Norte E.S.E. en Bogotá, Colombia, entre el 2019 y el 2020.

La plataforma MIPRES es la herramienta virtual de prescripción del Ministerio de Salud y Protección Social del gobierno colombiano, mediante la cual se autoriza la dispensación de medicamentos de alto costo financiados con recursos públicos del Sistema Nacional de Seguridad Social ^15^.

### Muestreo y recolección de datos

Se utilizó un método de muestreo por conveniencia para seleccionar la información de la base de datos MIPRES de pacientes a quienes se les recetó toxina botulínica en la Unidad de Servicios de Salud Simón Bolívar. Posteriormente, se obtuvieron las historias clínicas del sistema electrónico de información de la institución.

Para recopilar los datos necesarios, se creó un instrumento mediante el s*oftware* Microsoft Excel™. Se extrajeron datos sociodemográficos, clínicos y farmacoepidemiológicos de la plataforma MIPRES y de la historia clínica de los pacientes.

### Variables

Se consideraron las siguientes variables: sexo, edad, antecedentes farmacológicos, tipo de toxina botulínica utilizada, dosis empleada, vía de administración, especialidad correspondiente a la prescripción, diagnóstico para el que se indicó su uso y concordancia entre el diagnóstico y las indicaciones estipuladas por el Invima para la toxina botulínica.

Para efectos de este estudio, la “administración efectiva” del medicamento se definió como el registro de la prescripción del medicamento en el sistema MIPRES y la administración documentada de la toxina botulínica al paciente, respaldada por la historia clínica en la que se describa la aplicación del medicamento.

Para caracterizar el riesgo de interacciones farmacológicas, cada medicamento consignado en los antecedentes farmacológicos de los pacientes se clasificó según el sistema de clasificación ATC (*Anatomical Therapeutic Chemical*) ^16^. El riesgo de interacción se determinó con la herramienta de búsqueda de interacciones entre medicamentos Lexicomp *Drug Interactions*, que hace parte de la plataforma *UpToDate*^17^. La carga anticolinérgica se estimó con la calculadora ABC (*Anticholinergic Burden Calculator*) ^18^. Se ingresaron los antecedentes farmacológicos utilizados en el momento de la prescripción de la toxina botulínica y reportados en la historia clínica de cada paciente. Los pacientes se clasificaron según la puntuación obtenida en la herramienta ABC como: “sin carga anticolinérgica” (puntuación ABC = 0), “posible carga anticolinérgica” (puntuación ABC = 1) y “carga anticolinérgica definitiva” (puntuación ABC ≥ 2).

### Análisis descriptivo

Las variables cuantitativas, ordinales y nominales, se describieron utilizando proporciones. Después de realizar la prueba de normalidad de Kolmogorov-Smirnov, se identificó una distribución no paramétrica en las variables continuas, por lo tanto, estas se presentaron mediante medianas y rangos intercuartílicos (RIC).

### Análisis exploratorio

Se hizo un análisis bivariado exploratorio para comparar las diferencias entre los subgrupos de las variables de interés. Se aplicaron las pruebas de ji al cuadrado o la U de Mann-Whitney, según correspondiera. Se consideró como significancia estadística a un valor de p < 0,05. Para los análisis estadísticos se utilizó el *software* JAMOVI, versión 2.2.5.

### Consideraciones éticas

Este estudio fue clasificado como una investigación de riesgo mínimo y fue aprobado por el Comité de Ética Institucional de la Subred Norte en Bogotá, Colombia, bajo el número de protocolo SNCEI-160 del 1 ° de julio del 2021.

## Resultados

Se analizaron 197 pacientes con prescripción de toxina botulínica, con una mediana de edad de 59 años (rango intercuartílico, RIC: 33 a 69). Entre ellos, 134 (68 %) eran mujeres. La mediana de edad de las mujeres fue de 60 años (RIC: 48 a 69), mientras que, para los hombres, la mediana de edad fue de 42 años (RIC: 42 a 64,5). La edad mínima y la máxima a las que se administró la toxina botulínica, fueron de 5 y 89 años, respectivamente. Del total, 10 (5,1 %) pacientes eran menores de 18 años y 64 (32,5 %) eran mayores de 65 años.

De las 197 prescripciones registradas en la plataforma MIPRES, hubo administración efectiva de la toxina botulínica en 81 casos (41,1 %). No se identificaron diferencias estadísticamente significativas en cuanto al sexo (p = 0,203) o la edad (p = 0,202). De los 81 casos registrados con administración efectiva, el 63 % (n = 51) fueron atendidos por neurología, el 13,6 % (n = 11) por medicina física y rehabilitación, el 7,4 % (n = 6) por cirugía maxilofacial, el 4,9 % (n = 4) por oftalmología al igual que por otorrinolaringología, el 2,5 % (n = 2) por ortopedia así como por urología, y el 1,12% (n = 1) por dermatología.

### Características de prescripción de la toxina botulínica

Todas las preparaciones de toxina botulínica analizadas fueron de tipo A. La mediana de la dosis empleada fue de 200 unidades internacionales (UI (RIC: 100 a 300), con una dosis mínima de 5 UI y una dosis máxima de 2.000 UI. La mediana de la dosis utilizada fue significativamente mayor en hombres (300 UI) que en mujeres (100 UI) (p < 0,001). 

Las vías de administración más empleadas fueron la intramuscular (n = 143; 72,6 %; IC _95 %_: 66,0 a 78,3), la intradérmica (n = 34; 17,3 %; IC _95 %_: 12,6 a 23,2) y la subcutánea (n = 4; 2,0 %; IC _95 %_: 0,8 a 5,1). El resto de las vías representó el 8,1 % (IC _95 %_: 5,1 a 12,8) de los casos analizados e incluyeron la intravesical (n = 6; 3,0 %; IC _95 %_: 1,4 a 6,5), la subdérmica (n = 5; 2,5 %; IC _95 %_: 1,1 a 5,8 %), la intrafaríngea (n = 3; 1,5 %; IC _95 %_: 0,5 a 4,4), la intralinfática (n = 1; 0,5 %; IC _95 %_: 0,1 a 2,8) y la intracordal o intralaríngea (n = 1; 0,5 %; IC _95 %_: 0,1 a 2,8).

**Figura 1.**
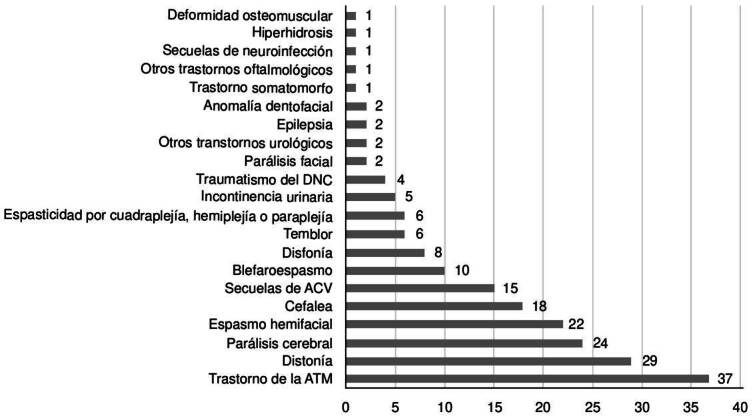
Prescripciones totales de toxina botulínica por diagnóstico (n = 197) SNC: sistemanervioso central; ACV: accidente cerebrovascular; ATM: articulación temporomandibular

Se identificaron 21 diagnósticos para los que se prescribió toxina botulínica (figura 1). Los trastornos de las articulaciones temporomandlbulares fueron más frecuentes en las mujeres (p ≤ 0,006) y la parálisis cerebral espástica fue más frecuente en los hombres (p ≤ 0,001). Solo 10 prescripciones (5,1 %) correspondieron a pacientes menores de 18 años. En este grupo, el 90 % se Indicó para el tratamiento de parálisis cerebral infantil y el 10 %, para Incontinencia urinaria por estrés.

Del total de prescripciones, 182 (92,4 %) fueron para indicaciones aprobadas según el Invima (cuadro 2), mientras que 15 (7,6 %) fueron para usos no autorizados (cuadro 3). La mediana de la dosis de los pacientes que recibieron la toxina botulínica por indicaciones aprobadas, fue menor (200 UI) que la de los pacientes que la recibieron por indicaciones no aprobadas (300 UI) (p ≤ 0,007). La edad (p = 0,351) y el sexo (p = 0,065) no tuvieron ningún impacto en estos dos tipos de prescripciones.

**Cuadro 2.** Prescripción de la toxina botulínica para usos aprobados por el INVIMA por condición, según la nomenclatura de la 10^a^ edición de la Clasificación Internacional de Enfermedades (CIE-10)

N°	CIE-10	Nombre	Número de pacientes		IC _95%_		Frecuencia acumulada (%)	
			n	%			
1	K076	Trastornos de la articulación temporomaxilar	37	18,8	13,94	24,81	18,8
2	G513	Espasmo hemifacial clónico	22	11,2	7,49	16,33	29,9
3	G800	Parálisis cerebral espástica	20	10,2	6,67	15,16	40,1
4	G249	Distonía no especificada	18	9,1	5,86	13,98	49,2
5	G439	Migraña no especificada	14	7,1	4,28	11,57	56,3
6	G245	Blefarospasmo	10	5,1	2,78	9,09	61,4
7	R490	Disfonía	8	4,1	2,07	7,81	65,5
8	G248	Otras distonías	8	4,1	2,07	7,81	69,5
9	G250	Temblor esencial	6	3,0	1,40	6,48	72,6
10	1698	Secuelas de otras enfermedades cerebrovasculares y de las no especificadas	4	2,0	0,79	5,10	77,2
11	G442	Cefalea debida a tensión	3	1,5	0,52	4,38	80,2
12	G809	Parálisis cerebral infantil sin otra especificación	3	1,5	0,52	4,38	81,7
13	G821	Paraplejia espástica	2	1,0	0,28	3,63	82,7
14	N318	Otras disfunciones neuromusculares de la vejiga	2	1,0	0,28	3,63	83,8
15	G824	Cuadriplejia espástica	2	1,0	0,28	3,63	84,8
16	T905	Secuelas de traumatismo intracraneal	2	1,0	0,28	3,63	85,8
17	K079	Anomalía dentofacial no especificada	2	1,0	0,28	3,63	86,8
18	T913	Secuelas de traumatismo de la médula espinal	2	1,0	0,28	3,63	87,8
19	G811	Hemiplejía espástica	2	1,0	0,28	3,63	88,8
20	G510	Parálisis de Bell	1	0,5	0,09	2,82	89,8
21	1663	Oclusión y estenosis de arterias cerebelosas	1	0,5	0,09	2,82	90,9
22	G253	Mioclonía	1	0,5	0,09	2,82	91,4
23	M210	Deformidad en valgo no clasificada en otra parte	1	0,5	0,09	2,82	92,9
24	G242	Distonía idiopática no familiar	1	0,5	0,09	2,82	93,4
25	G244	Distonía bucofacial idiopática	1	0,5	0,09	2,82	93,9
26	G519	Trastornos del nervio facial no especificado	1	0,5	0,09	2,82	94,4
27	R610	Hiperhidrosis localizada	1	0,5	0,09	2,82	94,9
28	R32X	Incontinencia urinaria no especificada	1	0,5	0,09	2,82	95,9
29	N394	Otras incontinencias urinarias especificadas	1	0,5	0,09	2,82	97,0
30	G241	Distonía idiopática familiar	1	0,5	0,09	2,82	97,5
31	G440	Síndrome de cefalea en racimos	1	0,5	0,09	2,82	98,0
32	N393	Incontinencia urinaria por tensión	1	0,5	0,09	2,82	98,5
33	N319	Disfunción neuromuscular de la vejiga no especificada	1	0,5	0,09	2,82	99,0
34	G808	Otros tipos de parálisis cerebral infantil	1	0,5	0,09	2,82	99,5
35	N310	Vejiga neuropática no inhibida, no clasificada en otra parte	1	0,5	0,09	2,82	100,0

**Cuadro 3.** Prescripción de la toxina botulínica para indicaciones no aprobadas (*off-label*) por el INVIMA por condición, según la nomenclatura de la 10a edición de la Clasificación Internacional de Enfermedades (CIE-10)

N°	CIE-10 Diagnóstico		N° pacientes n (%)	Especialidad prescriptora
1	I66.8	Otro infarto cerebral especificado	5 (2,5)	Medicina física y rehabilitación
2	I67.9	Enfermedad cerebrovascular no especificada	3(1,5)	Medicina física y rehabilitación
3	I64.X	Accidente cerebrovascular no especificado, como hemorragia o infarto	1 (0,5)	Neurología
4	I66.3	Oclusión y estenosis de arterias precerebrales, múltiples y bilaterales	1 (0,5)	Neurología
5	I67.8	Otras enfermedades cerebrovasculares especificadas	1 (0,5)	Neurología
6	G40.9	Epilepsia no especificada	1 (0,5)	Neurología
7	H57.8	Otros trastornos especificados del ojo y sus anexos	1 (0,5)	Oftalmología
8	G04.8	Otras encefalitis y encefalomielitis	1 (0,5)	Neurología
9	F45.8	Otros trastornos somatomorfos	1 (0,5)	Neurología

La toxina botulínica fue prescrita por ocho especialidades diferentes (cuadro 4). La que más frecuentemente prescribió toxina botulínica para indicaciones no aprobadas por Invima, fue medicina física y rehabilitación (8/15; 53,3 %; p ≤ 0,001), seguida por neurología (6/15; 40,0 %; p = 0,588) y oftalmología (1/15; 6,6 %; p = 0,556). No se observaron diferencias estadísticamente significativas en el riesgo de interacciones farmacológicas entre los pacientes que recibieron toxina botulínica por indicaciones aprobadas y aquellos que lo hicieron por indicaciones no aprobadas (p = 0,708).

**Cuadro 4.** Especialidades prescriptoras de toxina botulínica

Especialidad	Pacientes n (%)
Neurología	92 (46,7)
Cirugía maxilofacial	39(19,8)
Medicina física y rehabilitación	35 (17,8)
Oftalmología	10(5,1)
Otorrinolaringología	8(4,1)
Urología	7 (3,6)
Ortopedia	5 (2,5)
Dermatología	1 (0,5)

### Riesgo de interacciones farmacológicas

El consumo concomitante de otros medicamentos fue reportado por 163 (82,7 %) pacientes, con un rango de 1 a 16 medicamentos utilizados. Según la clasificación ATC, el 31,2 % de esos antecedentes farmacológicos involucraba medicamentos que actúan sobre el sistema nervioso central, el 28,3 %, sobre el sistema cardiovascular, el 15,2 % afectaban el tubo digestivo y el metabolismo, y el 6,3 % implicaba medicamentos hormonales sistémicos (excluyendo las hormonas sexuales). Se detectaron riesgos de interacciones farmacológicas con la toxina botulínica en 61 (30,9 %) pacientes con antecedentes farmacológicos positivos.

El riesgo de interacciones farmacológicas no se vio afectado por el sexo (p = 0,136) o la edad (p = 0,178). Tampoco se observaron diferencias estadísticamente significativas en su frecuencia entre pacientes mayores de 65 años (16/64; 25 %) y menores de 65 años (46/133; 33,8 %) (p = 0,209).

Al calcular la carga anticolinérgica, 34 (17,3 %) pacientes no tenían carga, 28 (14,2 %) tenían una posible carga anticolinérgica y 101 (51,3 %) tenían una carga anticolinérgica definitiva. La edad no se correlacionó con un aumento de la carga anticolinérgica (p = 0,412). Sin embargo, la presencia de una carga anticolinérgica posible o definida se asoció de forma estadísticamente significativa con un mayor riesgo de interacciones farmacológicas (p ≤ 0,001).

## Discusión

Las características sociodemográficas de la población estudiada son esenciales para entender los patrones de prescripción y acceso a la toxina botulínica. En la cohorte estudiada, la mayoría de los pacientes tratados fueron mujeres con una mediana de edad de 60 años. Sin embargo, no se encontraron diferencias estadísticamente significativas en la administración efectiva del medicamento según sexo o edad. Estos datos sugieren que, a pesar de las barreras de acceso, la toxina botulínica tiene un uso amplio y variado en diferentes grupos demográficos en el contexto de la salud pública.

Alrededor del 75 % de la población evaluada recibió prescripciones de toxina botulínica para uso intramuscular. Las vías autorizadas de administración por el registro de salud en Colombia son: intramuscular, intradérmica y subcutánea. Otras vías de administración descritas, como intralaríngea o intracordal, corresponden a la inyección intramuscular de la toxina en las cuerdas vocales para el tratamiento de la disfonía espasmódica ^19^; por su parte, la inyección intravesical se administra en los músculos de la vejiga para el tratamiento de la incontinencia urinaria ^20^. En los pacientes del presente estudio, se practicaron una aplicación intracordal y seis intravesicales. Durante el período analizado, no se registró ninguna administración intramuscular en el tubo digestivo, a pesar de que esta vía está descrita en la literatura ^21^.

En la presente investigación, ocho especialidades diferentes prescribieron toxina botulínica (cuadro 4). Más de dos tercios de las prescripciones correspondieron a neurología y cirugía maxilofacial. Otras especialidades médicas y quirúrgicas descritas como prescriptoras fueron oftalmología, neuropediatría, fisioterapia, urología, gastroenterología, otorrinolaringología, ortopedia, ginecología, reumatología, dermatología, medicina estética, y cirugía plástica ^3^^,^^22^. Solo un paciente recibió prescripción de la toxina botulínica para un trastorno somatomorfo, que luego se interpretó como un error de tipificación del diagnóstico (figura 1). Sin embargo, la literatura ha descrito los beneficios potenciales de la toxina botulínica en condiciones psiquiátricas, tales como depresión y trastorno límite de la personalidad, así como en complicaciones relacionadas con medicamentos psicotrópicos, como sialorrea inducida por neurolépticos y discinesias tardías ^23^^-^^25^.

La toxina botulínica se aplicó para distintas indicaciones en el presente estudio. Aunque los trastornos de la articulación temporomandibular fueron el diagnóstico más frecuente al momento de la administración de la toxina, la mayoría de las prescripciones estaban indicadas para condiciones neurológicas (figura 1), lo que se correlaciona con que neurología fuera la especialidad en la cual más se prescribió el medicamento. Asimismo, se pudo observar que el 92,4 % de las prescripciones de la toxina botulínica concuerdan con las indicaciones recomendadas por la autoridad reguladora de medicamentos en Colombia. Algunas de las prescripciones para indicaciones no aprobadas podrían resultar de errores en la entrada de datos o de los sistemas de prescripción.

En la literatura científica se describe una prevalencia entre el 0,4 % y el 2,6 % de reacciones adversas asociadas con el uso de la toxina botulínica. Estas reacciones pueden incluir síntomas como debilidad muscular, fatiga, disfagia, xerostomía, náuseas y cefalea, entre otros ^26^. Se recomienda hacerle seguimiento al paciente durante la administración de la toxina, especialmente, cuando se aplica de manera concomitante con aminoglucósidos, bloqueadores neuromusculares o medicamentos anticolinérgicos ^27^. Debido a su actividad anticolinérgica, la toxina botulínica se debe usar con precaución en pacientes con antecedentes de glaucoma de ángulo cerrado ^28^^,^^29^.

El uso de medicamentos con gran carga anticolinérgica se ha asociado con un mayor riesgo de fractura de cadera en pacientes mayores de 60 años ^30^, deterioro cognitivo, demencia y muerte ^31^^,^^32^.

En el presente estudio, el 32,5 % de la población era mayor de 65 años, quienes no presentaron diferencias estadísticamente significativas respecto a los menores de 65 años en cuanto a la carga anticolinérgica (p = 0,412) o el incremento en el riesgo de interacciones farmacológicas (p = 0,209). Sin embargo, debe considerarse que el número de pacientes menores de 65 años fue más del doble que el de los mayores de 65 años, lo que podría producir un sesgo en el análisis comparativo.

Por otra parte, se encontraron riesgos de interacciones farmacológicas con medicamentos que tienen efectos anticolinérgicos potenciales en casi un tercio de los pacientes, con una carga anticolinérgica posible o definitiva en el 65,5 % de los casos, lo que en teoría los predispone a resultados desfavorables. Por lo tanto, es necesario administrar dichos medicamentos de acuerdo con las directrices establecidas e implementar vías de control rigurosas para mitigar el riesgo.

En España, la toxina botulínica es el segundo medicamento más utilizado para indicaciones no aprobadas, después del rituximab ^33^. En Colombia, en el presente estudio, se encontró que el 7,6 % de las prescripciones de la toxina botulínica fueron para usos no aprobados por el Invima. De este porcentaje, el 60 % de las aplicaciones de la toxina botulínica se administró en pacientes con antecedentes de accidente cerebrovascular o condiciones relacionadas (cuadro 3). Al revisar las historias clínicas, se dedujo que la indicación principal era, probablemente, el tratamiento de las complicaciones asociadas con la espasticidad posterior al accidente cerebrovascular-un uso descrito en la literatura ^34^-y no con el accidente cerebrovascular *per se*.

Esta discrepancia puede deberse a la selección de un diagnóstico incorrecto en los sistemas de información, ya que la décima versión de la CIE-10 carece de un código específico para la espasticidad posterior al accidente cerebrovascular. En consecuencia, la ausencia de códigos de diagnóstico específicos en dicha clasificación dificulta que los profesionales de la salud ingresen los diagnósticos que realmente corresponden a las indicaciones aprobadas del medicamento.

La imprecisión en los diagnósticos y en los registros de administración de la toxina botulínica, se asocian con la falta de claridad sobre la demanda real de este tratamiento. Como no se registran adecuadamente las indicaciones clínicas auténticas de la prescripción, los entes reguladores encargados de su vigilancia y control subestiman la necesidad de la toxina. En consecuencia, los recursos financieros destinados al acceso y distribución de la toxina botulínica se asignan con parámetros inexactos, lo que limita su oferta y repercute directamente en el acceso al tratamiento.

En Colombia, la tasa de cobertura de atención médica alcanza el 94,4 % de la población. Sin embargo, aproximadamente, el 21 % de las personas no puede acceder efectivamente a estos servicios ^35^ y alrededor del 7,3 % de las dificultades de acceso se deben a que no se autoriza la entrega de suministros o la dispensación de medicamentos ^36^. El Ministerio de Salud y Protección Social de Colombia ha desarrollado estrategias, como la plataforma MIPRES, para reducir las barreras de acceso a tecnologías sanitarias de alto costo ^37^, entre las que se incluye la toxina botulínica.

Sin embargo, en el presente estudio, solo el 41,1 % de los individuos a quienes se les prescribió el medicamento mediante la plataforma, realmente lo recibieron. La mayoría (63,0 %) de las prescripciones correspondieron al área de la neurología, para tratar condiciones como espasticidad, blefaroespasmo, espasmo hemifacial, distonías y migraña crónica. Otras especialidades con registros de administración efectiva fueron: medicina física y rehabilitación (13,6 %), para el tratamiento de complicaciones asociadas con la espasticidad posterior a un accidente cerebrovascular y las derivadas de la parálisis cerebral infantil; cirugía maxilofacial (7,4 %), para el diagnóstico de trastorno de la articulación temporomaxilar; y otorrinolaringología (4,9 %), para el tratamiento de la disfonía, entre otras condiciones (cuadro 5).

**Cuadro 5.** Administración efectiva de toxina botulínica

CIE-10	Diagnóstico	Prescripciones (n = 81)	Especialidad prescriptora (n)
G048	Otras encefalitis, mielitis y encefalomielitis	1	Neurología (1)
G241	Distonía idiopática familiar	1	Neurología (1)
G242	Distonía idiopática no familiar	1	Neurología (1)
G245	Blefarospasmo	3	Neurología (1) Oftalmología (2)
G245	Hiperhidrosis localizada	2	Oftalmología (1) Dermatología (1)
G248	Otras distonías	4	Neurología (4)
G249	Distonía no especificada	10	Neurología (10)
G250	Temblor esencial	4	Neurología (4)
G253	Mioclonía	1	Neurología (1)
G439	Migraña no especificada	7	Neurología (7)
G442	Cefalea debida a tensión	2	Neurología (2)
G513	Espasmo hemifacial clónico	15	Neurología (15)
G519	Trastornos del nervio facial no especificados	1	Neurología (1)
G800	Parálisis cerebral espástica	5	Ortopedia (2) Medicina física y rehabilitación (3)
G808	Otros tipos de parálisis cerebral infantil	1	Medicina física y rehabilitación (1)
G809	Parálisis cerebral infantil sin otra especificación	1	Medicina física y rehabilitación (1)
H578	Otros trastornos especificados del ojo y sus anexos	1	Oftalmología (1)
I64X	Accidente vascular encefálico agudo no especificado, como hemorrágico o isquémico	1	Neurología (1)
1663	Oclusión y estenosis de arterias cerebelosas	1	Neurología (1)
1668	Oclusión y estenosis de otras arterias cerebrales	2	Medicina física y rehabilitación (2)
1678	Otras enfermedades cerebrovasculares especificadas	1	Neurología (1)
1679	Enfermedad cerebrovascular no especificada	1	Medicina física y rehabilitación (1)
1698	Secuelas de otras enfermedades cerebrovasculares y de las no especificadas	1	Medicina física y rehabilitación (1)
K076	Trastornos de la articulación temporomaxilar	6	Cirugía maxilofacial (6)
N310	Vejiga neuropática no inhibida, no clasificada en otra parte	1	Urología (1)
N318	Otras disfunciones neuromusculares de la vejiga	1	Urología (1)
R490	Disfonía	4	Otorrinolaringología (4)
T905	Secuelas de traumatismo intracraneal	1	Medicina física y rehabilitación (1)
T913	Secuelas de traumatismo de la médula espinal	1	Medicina física y rehabilitación (1)

El bajo porcentaje de pacientes que efectivamente recibieron la toxina botulínica sugiere la existencia de barreras administrativas relacionadas con el uso de las plataformas de prescripción y administración de medicamentos, y con la especialidad prescriptora. Se espera que el acceso mejore sustancialmente con la promulgación de la Resolución 2808 de 2022, que incluyó la toxina botulínica como un medicamento que debe ser accesible sin autorización previa mediante la plataforma MIPRES ^38^.

A pesar de los avances alcanzados en los últimos años dentro del sistema de salud colombiano, aún persisten deficiencias serias de vigilancia y cobertura. Existe una desarticulación entre las entidades reguladoras y los sistemas de vigilancia en salud -como el Invima y el Sistema de Inspección y Control en Salud ^14^-, lo que conlleva procesos de supervisión poco efectivos en las instituciones prestadoras de servicios. Por ende, muchas de las acciones ejecutadas no responden a las necesidades reales de las instituciones y no garantizan el cumplimiento de los derechos de los pacientes. Una articulación de estas entidades es necesaria para implementar nuevas regulaciones que sean capaces de mitigar las limitaciones del acceso al sistema.

Este estudio tiene varias limitaciones. Primero, dado el carácter retrospectivo de los datos, es común que no se especifique el tipo de toxina botulínica utilizada. Esta falta de información puede resultar en una posible incongruencia entre la prescripción y la indicación, subestimando potencialmente la precisión de la prescripción. Segundo, las indicaciones aprobadas por el Invima están disponibles de forma descriptiva y no según los diagnósticos consignados en la CIE-10, por lo que el análisis de la información puede estar sujeto a sesgos; y, tercero, el diseño del estudio no permitió una evaluación cronológica para establecer si la administración de la toxina botulínica coincidió temporalmente con el consumo de medicamentos con potencial interacción.

Por lo tanto, no se pudo estimar el riesgo de reacciones adversas al medicamento asociadas con una posible sinergia anticolinérgica. Además, en este estudio se experimentó un sesgo de caso atípico, ya que el periodo analizado incluyó un año de pandemia por SARS-CoV-2, lo que pudo afectar el panorama del uso y disponibilidad de la toxina botulínica en las instituciones de salud. Durante el 2020, los pacientes no consultaban por motivos diferentes a los relacionados con el coronavirus, lo que generó un descenso en la atención de casos que requerían tratamiento con toxina botulínica.

Según la Organización Panamericana de Salud (OPS), entre el momento en que se detectó el primer caso de COVID-19 en la región de las Américas en enero del 2020 y el 4 de enero del 2023, se notificaron 186’265.607 casos positivos, entre ellos, 2’891.057 de muertes ^39^. Por esta razón, todos los esfuerzos, los recursos y el personal fueron redireccionados a controlar esas cifras, lo que redujo aún más la cantidad de atención destinada a otros tratamientos. Además, se presentó un desabastecimiento de medicamentos, lo que incrementó la brecha de acceso a terapias no relacionadas con COVID-19.

Este estudio proporciona un panorama general sobre el uso y el perfil farmacológico de la toxina botulínica en Colombia, lo que permitió establecer una base para consolidar su aplicación en condiciones médicas no cosméticas. Este enfoque no solo favorece su incorporación en la práctica clínica, sino que también impacta significativamente la salud pública, al promover la formulación de regulaciones orientadas a un uso más seguro y a la reducción de barreras de acceso para los pacientes.

Futuros estudios deben centrarse en estimar con precisión la sinergia anticolinérgica para abordar riesgos potenciales asociados con las interacciones farmacológicas y las complicaciones derivadas de una gran carga anticolinérgica. Además, es crucial que los investigadores especifiquen el tipo exacto de toxina botulínica utilizada para garantizar su eficacia clínica. Por último, se requieren estudios prospectivos que evalúen su efectividad médicamente en indicaciones no aprobadas y, así, regular tales usos ante las agencias nacionales e internacionales.
